# Peculiarities of Ringworm and Its Treatment

**Published:** 1880-03

**Authors:** 


PECULIARITIES OF RINGWORM AND ITS TREATMENT.

In the *Lodon Lancet* for January, Dr. Liveing directs attention to the peculiarities of ringworm. He notes the prevalence of this disease in England and France, and its comparative infrequency in Germany. In ringworm of the scalp, which is far more important than that of the body, the diagnosis is often difficult. As to its communicability, he says it is so until it is quite cured; indeed as long as scurfy spots remain, even though the hair be growing freely, it may be propagated. It is far more contagious when it first appears before any remedies have been applied than it is while under treatment. The best remedy to prevent its spreading among children is the carbolized glycerine of the British Pharmacopœia, either pure or diluted with a little more glycerine; it should be well rubbed over the scalp every morning. This acts in two ways; (1) the carbolic acid produces its usual effect on organized matter; and (2) the glycerine prevents particles of scurf from being dispersed. In addition to this the carbolized glycerine has a distinct curative effect.

As to the treatment of this troublesome malady, the remedies may be divided into two classes: (1) those which act by setting up sufficient inflammation in the skin to lead to the destruction of the disease; (2) those of a milder kind which act simply as antagonistic to the development of the Trichophyton tonsurans. To the former class belong such remedies as acetum cantharidis and strong acetic acid; to the latter, sulphur ointment, the white precipitate ointment, and sulphurous acid lotion. Many remedies combine these two properties; as for example, chrysophanic acid ointment, iodine liniment and strong carbolized glycerine.

How is the choice to be determined between these and many other remedies. *Strong remedies are* always contra-indicated in very young children. A little tincture of iodine painted on once a day for a few days, followed by the use of the white precipitate ointment is all that is necessary. In older children, stronger treatment must be used; it is very unwise to make a *large* sore place on the scalp, as it may give more trouble than the ringworm itself. If the disease is in the early stage, and consists of one or two circumscribed spots, cut the hair short all around the spots, and apply with a brush Costel's paste, acetum cancharides, or iodine liniment. A single painting with pure carbolic acid is thoroughly effective, but it is a strong remedy and gives some pain. It is very unwise to trust strong remedies in unskilled hands. When the disease extends over a large surface, use milder measures; as tincture of iodine of double strength painted on every day, followed if necessary by the nitrate of mercury ointment, diluted according to circumstances, or an ointment containing the red or white precipitate of mercury and sulphur, or the oleate of mercury (10 per cent). In certain cases goa powder, or chrysophanic acid ointment (thirty grains to the ounce) are very effective.

Ringworm is often associated with a generally unhealthy condition of the skin which is badly flourished. In such cases tonics, as iron and arsenic, are often useful. This is in accordance with the fact that many strictly local affections are influenced by general treatment.

